# Tunable optical nonlinearity of indium tin oxide for optical switching in epsilon-near-zero region

**DOI:** 10.1515/nanoph-2022-0306

**Published:** 2022-08-09

**Authors:** Kuen Yao Lau, Yuting Yang, Di Zhao, Xiaofeng Liu, Jianrong Qiu

**Affiliations:** College of Optical Science and Engineering and State Key Lab of Modern Optical Instrumentation, Zhejiang University, 310027, Hangzhou, China; School of Materials Science and Engineering, Zhejiang University, 310027, Hangzhou, China; Wuhan National Laboratory for Optoelectronics, Huazhong University of Science and Technology, 430074, Wuhan, China

**Keywords:** magnetron sputtering deposition, metal oxide semiconductor, optical nonlinearity, saturable absorption, thin film

## Abstract

The propagation of light in the epsilon-near-zero (ENZ) region of materials exhibits intriguing linear and nonlinear optical phenomenon that have been extensively exploited for a plethora of applications. Here, we show that the optical properties as well as the ENZ wavelength of magnetron-sputtered indium tin oxide (ITO) thin films could be judiciously engineered. The measurement of nonlinear optical properties reveals that the control of deposition conditions allows for the tuning of absorptive optical nonlinearity between saturable absorption and reverse saturable absorption. The ENZ wavelength for the ITO film is deduced as around 1553 nm. We obtain the highest third-order nonlinear absorption coefficient and imaginary part of third-order nonlinear susceptibility for the ITO thin film through Z-scan method as −50.56 cm/GW and ∼38 × 10^−14^ e.s.u. at 1050 nm, and −64.50 cm/GW and ∼45 × 10^−14^ e.s.u. at 1550 nm, respectively. We demonstrate further that the strong saturable absorption of the ITO thin film enables Q-switched pulse laser generation in ∼1050 and ∼1550 nm regions with tunable repetition rates and pulse energies. The present results suggest the great application potential of the ITO thin film in the field of nonlinear optical devices.

## 1 Introduction

The nonlinear optical response of matter to light is perturbative and it is inherently a weak effect. Most materials exhibit weak optical nonlinearity even under intense coherent illumination. A substantial development in nonlinear optics is the drastically change in refractive index of materials using a low-power optical field. Epsilon-near-zero (ENZ) materials are a new class of materials with massive reduction of dielectric permittivity, *ε* at the ENZ wavelength. For a given change in permittivity, Δ*ε*, the resultant change in refractive index, Δ*n* is provided for a lossless material by [Δ*n =* Δ*ε*/2*(*ε*)^1/2^] [1]. A higher Δ*n* denotes stronger nonlinear optical response, whilst *ε* becomes extremely small at the ENZ wavelength. Most metals and heavily doped metal oxide semiconductors with high concentration of free carries, i.e., indium tin oxide (ITO), have zero *ε* in the visible and near infrared wavelength regions. These ENZ materials have been demonstrated in various nonlinear optics applications, i.e., second-harmonic generation, third-harmonic generation, four-wave mixing and optical switching [2–7].

The strong optical nonlinearity is required for applications as optical switches, which, for instance, have been used to drive pulse generation in pulse lasers [8]. These optical switches are also known as saturable absorbers that make use of the strong and fast absorptive nonlinearity with a negative nonlinear absorption coefficient. Pulsed laser generates discrete packet of energy that can produce peak power far greater than its average power. In contrast to continuous-wave (CW) fiber lasers, pulsed fiber lasers generate nanosecond to femtosecond pulses that facilitates a vast range of laser precision microfabrication processes such as drilling, cutting, welding, marking, and micro-forming [9]. A pulsed laser has the ability to store and release energy very rapidly thus delivering an intense electromagnetic energy with kilowatts to megawatts peak power. Apart from the laser precision microfabrication, the high peak power enables numerous nonlinear optical processes, which involves the interaction of more than one photon with matter at a time. Saturable absorption is among the nonlinear optical processes which exhibits decreased light absorption at higher optical fluence. Photo-bleaching is induced when the saturable absorption of a material occurs under intense illumination. The photo-bleaching could not only control the on/off switching status of a laser signal, but also converts the CW laser into pulsed laser. In general, all materials should have certain degree of saturable absorption properties, even the cleaning ingredients in the laboratory, alcohol, was employed as a saturable absorber for mode-locked fiber laser [10]. However, the saturation intensities of most materials are very high, which is sometimes higher than the optical damage threshold. Till now, there are only few materials, i.e., carbon nanotube [11], graphene [12], transition metal dichalcogenides [13], topological insulator [14], black phosphorus [15], and MXene [16] that show significant saturable absorption effect.

Here, we examined the linear and nonlinear optical properties of the ITO thin films synthesized by the magnetron-sputtering deposition (MSD) method. By using the Z-scan method, we investigated the third-order nonlinear absorption coefficient (*β*) and imaginary part of third-order nonlinear susceptibility (Im*χ*^(3)^) for ITO thin films, and observed strong dependence of NLO parameters on fabrication conditions. We obtained the highest *β* and Im*χ*^(3)^ of −50.56 cm/GW and ∼38 × 10^−14^ e.s.u. for the ITO thin film with the least flow rate of oxygen content under inert condition (O_2_:Ar ratio of 0.0) at 1050 nm. Moreover, the highest *β* and Im*χ*^(3)^ of −64.50 cm/GW and ∼45 × 10^−14^ e.s.u. for the ITO thin film were attained at 1550 nm. The ITO thin film with the optimized optical nonlinearity parameter was employed for the optical switching in a Q-switched pulsed fiber laser at ∼1 μm region for the first time, to the best of authors’ knowledge. In addition, the ITO-based switch also enables pulsed fiber laser generation with a single-pulse energy of 38.9 nJ at ∼1.5 μm wavelength. Our result reveals the feasibility to control the optical nonlinearity of metal oxide semiconductor and its application as optical switches for pulsed fiber laser applications.

## 2 Experimental section

### 2.1 Sample preparation

The ITO thin film was prepared via the MSD method. The ITO (In_2_O_3_ + SnO_2_ with purity of 99.99%) was employed as the sputtering target. This ITO target was pre-sputtered with pure argon gas for cleaning of the surface. The conditions for pre-sputtering process are background vacuum degree of 2.0 × 10^−3^ Pa, working pressure of 2.0 Pa, power of 80 W and argon gas flow rate of 80 mL/min. The quartz substrate with thickness of 0.5 mm was ultrasonically cleaned in a mixture of acetone, ethanol, and deionized water. Next, this quartz substrate was dried with nitrogen gas and placed horizontally in the sputtering chamber. The background vacuum degree and the direct current (DC) power applied to the ITO target were kept constant at 8.0 × 10^−4^ Pa and 80 W, respectively. Based on different ratio of O_2_:Ar supplied to the chamber, the sputtering process was conducted under the pressure of 0.6 Pa. During the sputtering process, the ITO thin film was deposited on the quartz substrate with fixed flow rate of argon gas as 40 mL/min. After completing the MSD process, the ITO samples were placed into a muffle furnace for annealing at 400 °C for an hour. Comparison was made for the ITO thin films before and after annealing process. The samples were named as IT-0.0, IT-0.5, and IT-8.0 for the sample before annealing with O_2_:Ar ratio of 0.0, 0.5 and 8.0, respectively. These samples were named as IT-0.0-400, IT-0.5-400, and IT-8.0-400 after annealing.

### 2.2 Material characterizations

Scanning electron microscope (SEM) images of the ITO samples were taken using a field-emission SEM (Hitachi SU-8010) operated at an accelerating voltage of 5 kV. X-ray diffraction patterns of the ITO samples were recorded using a SmartLab diffractometer with Cu K*α* radiation, step width of 0.02° and scanning speed of 5°/min. Raman spectra of the ITO samples were recorded with a Renishaw inVia confocal Raman system using 534 nm excitation laser for 10 s. Chemical composition of the ITO samples was determined by the Raman spectra and X-ray photoelectron spectroscopy (Thermo Scientific ESCALAB 250Xi). Reflection and absorption spectra of the ITO samples from 400 to 1600 nm were measured via the Hitachi UH-5700 UV-vis-NIR spectrophotometer with the help of an integrating sphere. In the reflectivity measurement, a BaSO_4_ plate was used as the reference for the collection of the baseline.

### 2.3 Z-scan measurement

The open-aperture Z-scan method was employed for the NLO absorption measurement of the ITO thin films. The excitation beam was generated by an optical parametric amplification laser (PH1-06-0200-02-10) with a pulse width of 50 fs and a repetition rate of 30 kHz. The collimated laser input was focused at the focus point, *z* = 0 with a convex lens with focal length of 12 mm. The focused laser spot diameters and laser fluences at both 1050 and 1550 nm excitation wavelength was measured as ∼73 μm and ∼120 GW/cm^2^, respectively. The ITO samples were positioned on a translational stage which was adjusted horizontally along the *z*-direction with total moving distance of 20 mm. The transmitted light intensities of the ITO samples were collected with a power detector (Thorlabs S121C).

### 2.4 Pulsed laser experiment

The IT-0.0 thin film deposited onto a thin quartz glass slide (thickness: 50 μm, size: 1.0 mm × 1.0 mm) was placed in between two fiber ferrules to form a saturable absorber. The schematic diagram of the ring-structured fiber laser cavity is illustrated in Figure S1. A section of rare-earth gain fiber, OFS YDF-350 and LIEKKI Er 110-4/125 were pumped by a 980 nm laser diode (LD) via a wavelength division multiplexer (WDM) in the ∼1.0 and ∼1.5 μm fiber lasers. An isolator ensures unidirectional signal propagation of the laser cavity. The output laser was measured through the 10% signal siphoned from a 90:10 optical coupler. The polarization state was adjusted by a polarization controller. All components are polarization insensitive which avoids the possibilities of pulse-induced nonlinear polarization rotation. At the initial stage, the laser cavity was examined without the saturable absorber. This is to validate that the laser pulses are solely contributed by the IT-0.0 saturable absorber.

## 3 Results and discussion

The microscopic characteristics of the ITO samples could be observed from the SEM images. The surface topography and thickness of the ITO thin films on quartz substrates were shown in Figures S2 and S3. The surface topography of the thin film is determined by the deposition techniques and parameters which influences its electrical, optical and mechanical properties. All the ITO thin films show closely-packed surface topography composed of grain profiles with average sizes of ∼0.1–1 μm before annealing and ∼0.1–0.3 μm after annealing. This difference in sample characteristics is mainly ascribed to the change in O_2_:Ar ratio during the sputtering process. All the ITO thin films are uniformly densified and firmly attached to the quartz substrate. There are no obvious macro-defects such as pinholes existing between the grains. From Figure S3, the thickness of all ITO thin films was measured as ∼2 μm.

**Figure 1:**
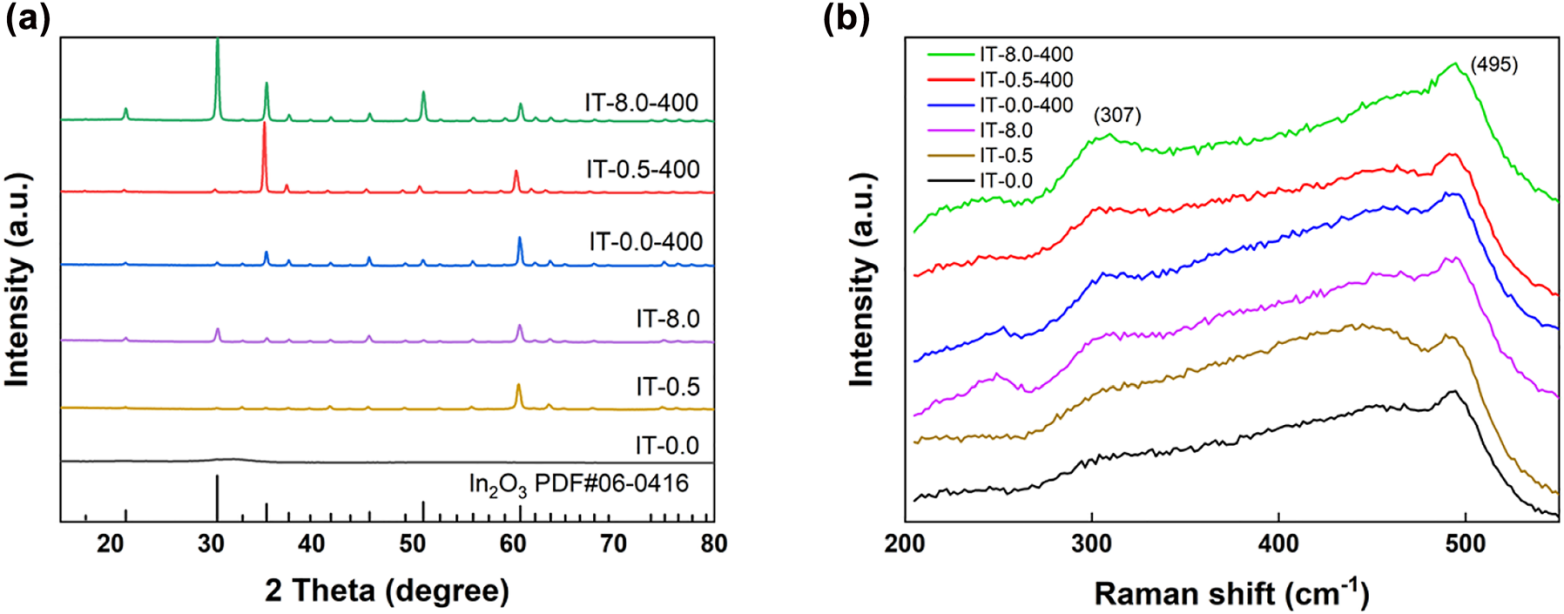
Optical characterization of ITO thin films. (a) XRD patterns and (b) Raman spectra of ITO thin films.

The phase compositions of the ITO thin films were identified with XRD patterns, as shown in Figure 1(a). Higher O_2_:Ar ratio contributes to higher crystallinity of the ITO thin film in the sequence of IT-8.0 > IT-0.5 > IT-0.0. All the observed diffraction peaks are assigned to the In_2_O_3_. The broad XRD pattern of IT-0.0 with 2*θ* at only 30.58° denotes the amorphous nature of this ITO sample, which is more brittle compared to IT-0.5 or IT-8.0. After annealing, the ITO thin films show stronger diffraction peaks. The diffraction peaks of 2*θ* at ∼30°, ∼35°, ∼51° and ∼60° correspond to the (222), (400), (440), and (622) planes of the In_2_O_3_. In order to further illustrate the structural properties of these ITO thin films, the Raman spectrum analysis was done for these samples in Figure 1(b). These ITO thin films show the Raman shift with characteristic peaks of 307 and 495 cm^−1^, whereas the 455 cm^−1^ peak denotes the quartz substrate [17–19]. All ITO thin films exhibit high phase purity and characteristic peaks of SnO_2_ are not present in the Raman spectra.

The chemical composition in the ITO thin films on quartz substrate was characterized by an X-ray photoelectron spectrum (XPS) as presented in Figure S4. Figure S4(a) shows the strongest peak of In-3d, as well as other peaks featuring the Sn-3d, O-1s, and In-3p. Figure S4(b) and (c) present the corresponding In-3d and Sn-3d peaks with high resolution extracted from the full XPS. While Raman spectrum reveals only a slight change in the peak position and intensity, the high resolution XPS spectra exhibit a clear shift of In 3d from ∼452 eV (In-3d_3/2_) to ∼444 eV (In-3d_5/2_) and Sn 3d from ∼495 eV (Sn-3d_3/2_) to ∼486 eV (Sn-3d_5/2_). The binding energies of In-3d_5/2_ and Sn-3d_5/2_ at 443.68 and 485.68 eV confirm the oxidation states of the In^3+^ and Sn^4+^, respectively [20].

**Figure 2:**
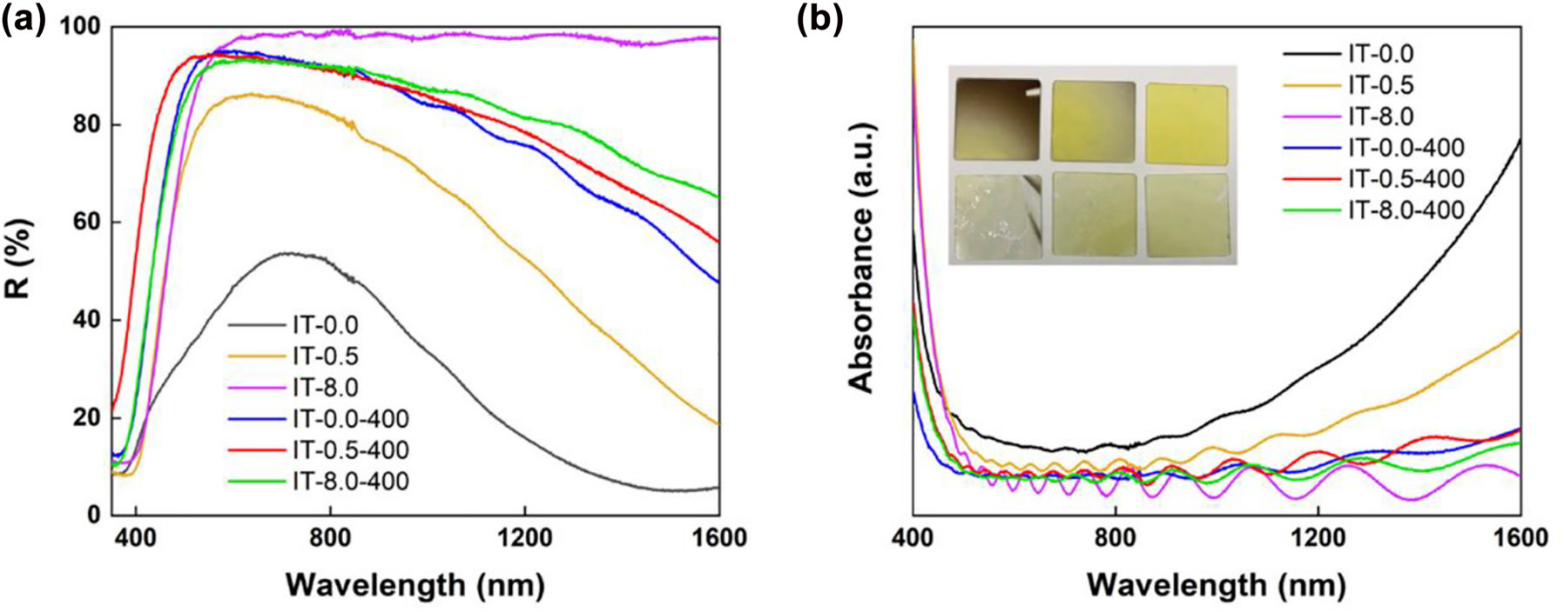
Optical characterization of ITO thin films. (a) Reflection and (b) absorption spectra of ITO thin film samples.

The linear optical properties of the ITO thin films were characterized through the UV-vis-NIR spectra from ∼350 to 1600 nm in Figure 2(a). Before annealing, the reflection of the ITO samples shows strong dependence on the O_2_:Ar ratio applied in the sputtering process. A higher oxygen ratio facilitates the elimination of oxygen vacancies in the films, which contributes to the loss of reflectance as observed across the visible and near-infrared (NIR) regions. For instance, the sample IT-8.0 shows almost no absorption from 500 to 1600 nm, which is in sharp contrast to sample IT-0.0 prepared in pure argon. After annealing in air, the reflectance curves for all the three samples are close as these samples are fabricated from the same target with the same composition. The absorption spectra of the ITO thin films were depicted in Figure 2(b). The introduction of higher oxygen deficiency during the sample preparation process, i.e., IT-0.0 and IT-0.5, induces stronger absorption in the NIR region, which agrees with the reflection spectra. This impact is less significant in the visible region. The inset in Figure 2(b) gives the photographs of all the ITO thin films. Darker color was observed for samples fabricated at lower O_2_:Ar ratio (from IT-8.0 to IT-0.0) and all the ITO samples become more transparent after annealing.

**Figure 3:**
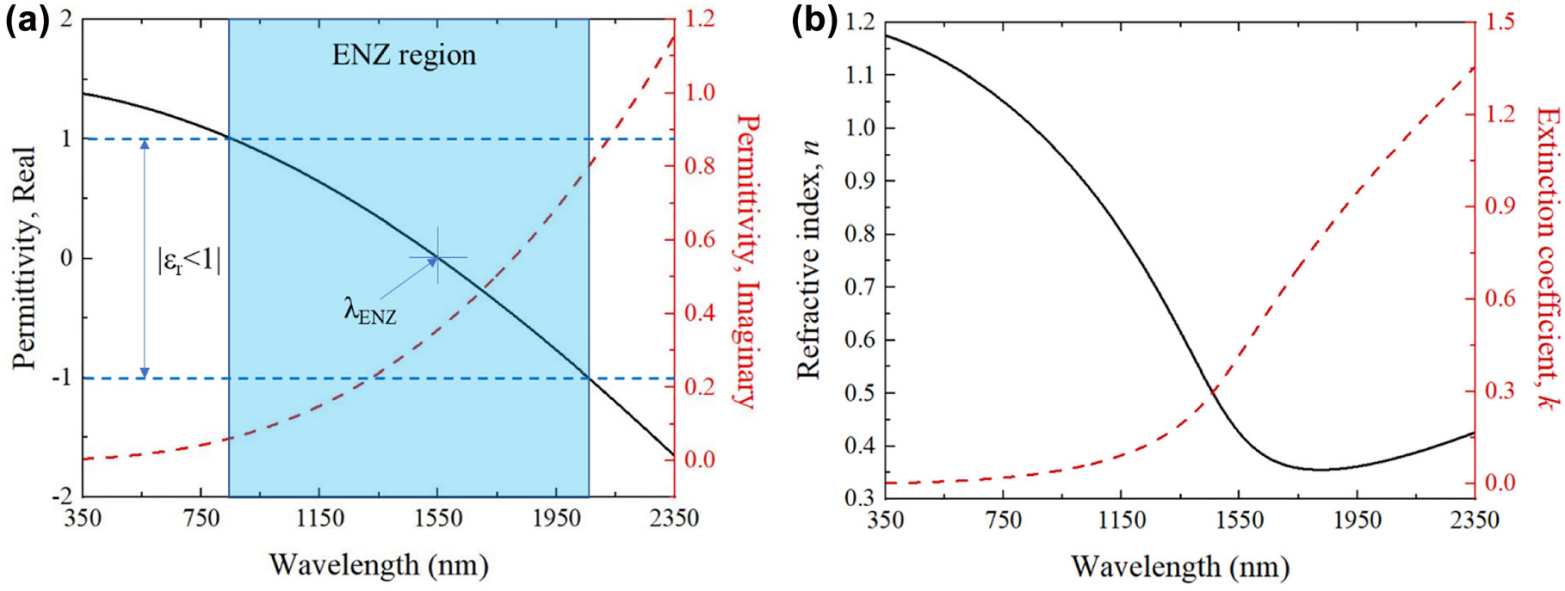
ENZ region characterization of ITO thin films. (a) The real and imaginary part permittivity (*ε*) of IT-0.0 calculated from Drude–Lorentz model. (b) The refractive index (*n*) and extinction coefficient (*k*) of IT-0.0.

The ENZ wavelength (*λ*_ENZ_) was calculated to be 1553 nm for sample IT-0.0 at the condition of real part of permittivity reaches zero (*ε*_r_ = 0), which is derived by fitting the reflectance curve using the Drude–Lorentz Model with the equations given in the Supporting Information. At the *λ*_ENZ_, the permittivity imaginary part (*ε*_*i*_) shows a value of 0.36, associated with the damping losses in the ITO. The ENZ region of the IT-0.0 (with |*ε*_r_ < 1|) was found to cover a spectral range from 855 to 2060 nm in Figure 3(a). This validates the high NLO response, i.e., high Δ*n* owing to the extremely small *ε*_r_ of the IT-0.0 in this NIR wavelength region covering the popular NIR wavelengths, i.e., ∼1060, ∼1550 and ∼2000 nm, which is attractive for a variety of NLO applications, such as optical switching and saturable absorption. The refractive index (*n*) and extinction coefficient (*k*) of the IT-0.0 was calculated according to the equation expressed in the Supporting Information.

**Figure 4:**
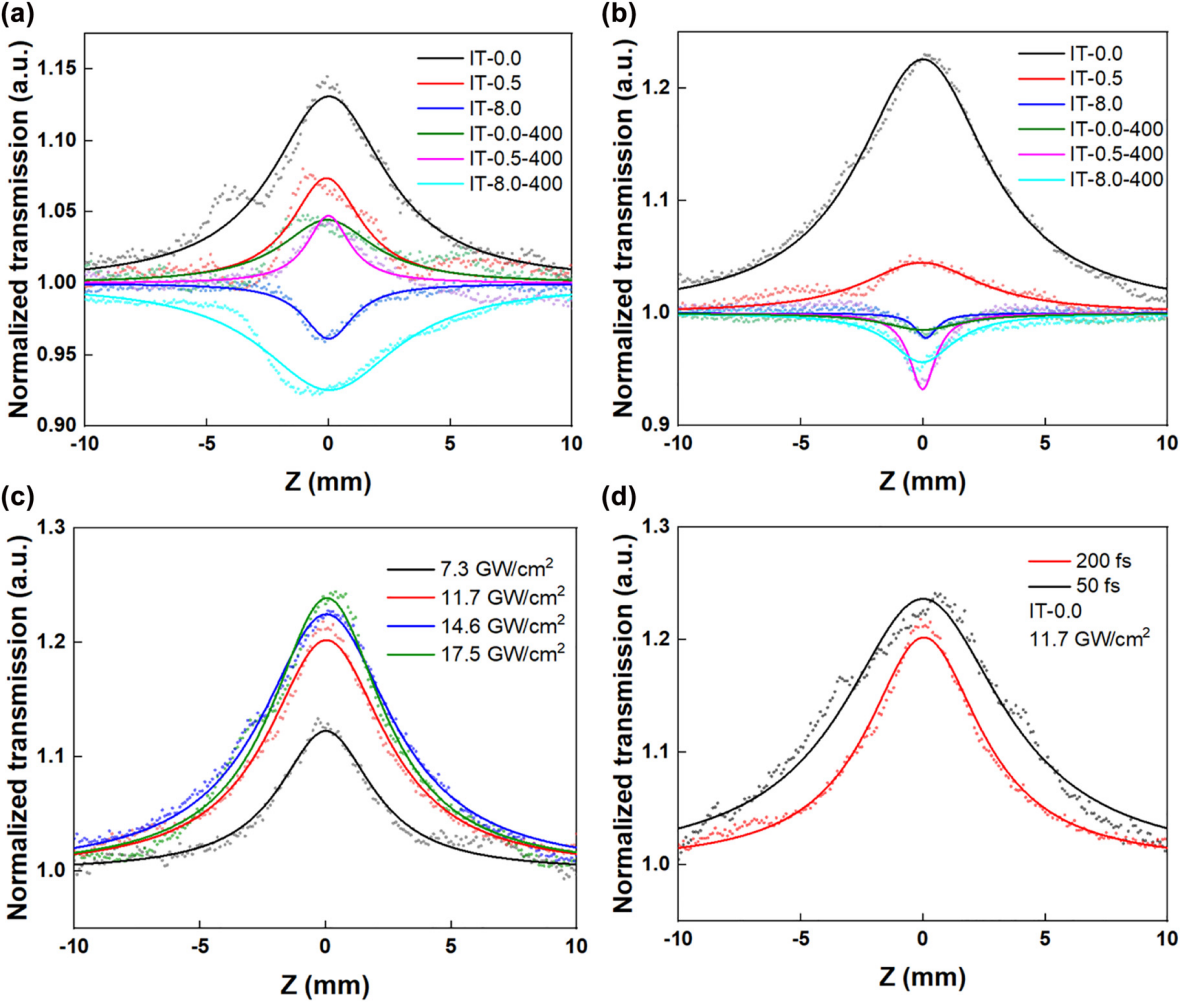
Z-scan curves of different ITO samples at (a) 1050 nm and (b) 1550 nm. Z-scan curves of IT-0.0 with different (c) excitation laser intensities and (d) pulse duration at 1550 nm.

The introduction of oxygen deficiency into the ITO thin film leads to reduced bandgap and changes in linear and nonlinear optical response. The Z-scan curves for different ITO samples are illustrated in Figure 4. The Z-scan measurement of different ITO samples was performed at the laser excitation wavelengths of 1050 and 1550 nm. The transmission reaches the positive plateau at the beam focus point (*z* = 0 mm) for samples of IT-0.0 and IT-0.5 due to saturable absorption, whereas a negative plateau in transmission is observed at the beam focus point for IT-8.0 due to reverse saturable absorption. The reverse saturable absorption could be employed for optical limiting application. At 1050 nm, IT-0.0, and IT-0.5 show a clear saturable absorption behavior, whereas IT-8.0 shows emergence of reverse saturable absorption after annealing. At 1550 nm, IT-0.0, and IT-0.5 show only reverse saturable absorption whereas the reverse saturable absorption for IT-8.0 becomes dominating after annealling. This tuning of nonlinear optical response could be better interpretted by characterizing the Hall effect properties of the ITO thin films [21]. The electrical properties of the ITO thin film were modified with higher electron mobility and lower free-carrier concentration after annealing process which leads to suppression of ground-state free-electrons bleaching. Mayer and Keilmann showed that the SA coefficient which correlates to the Im*χ*^(3)^ is proportional to the free-carrier concentration [22]. With reduced free-carrier concentration at high temperature, the saturable absorption effect becomes weakened whereas the reverse saturable absorption is strengthened. In addition, a lower O_2_:Ar ratio during the sample preparation induces stronger saturable absorption effect both at 1050 and 1550 nm. This also provides the feasibility to tailor the nonlinear optical absorption properties by controlling the growth atmosphere. At both the laser excitation wavelengths of 1050 and 1550 nm, IT-0.0 shows the highest transmission among the ITO samples in Figure 4(a) and (b). Therefore, the IT-0.0 was further investigated by optimizing the excitation laser intensities and pulse durations at 1550 nm. By increasing excitation laser intensities from 7.3 to 17.5 GW/cm^2^, the transmission of IT-0.0 increases along with higher laser intensities in Figure 4(c). By maintaining the laser intensities at 11.7 GW/cm^2^, the pulse duration of the excitation laser was adjusted as either 50 or 200 fs. The normalized transmission of IT-0.0 is higher when using shorter pulse duration in Figure 4(d).

The Z-scan measurement was conducted to deduce the nonlinear absorption coefficient (*β*) and imaginary part of third-order nonlinear susceptibility (Im*χ*^(3)^) for all ITO thin films. The Z-scan curves were fitted with nonlinear absorption model, whereas the *β* and Im*χ*^(3)^ for these ITO thin films were calculated according to the formulas given in Supporting information. The values of *β* and Im*χ*^(3)^ for the ITO samples at 1050 and 1550 nm were calculated and summarized in Table 1. The negative and positive values of *β* indicate the saturable absorption and reverse saturable absorption characteristics, respectively. Based on the calculation, the IT-0.0 exhibits the highest *β* of −50.56 cm/GW and Im*χ*^(3)^of ∼38 × 10^−14^ e.s.u. among the ITO samples at 1050 nm. In addition, the highest *β* of −64.5 cm/GW and Im*χ*^(3)^of ∼42 × 10^−14^ e.s.u. among the ITO samples were obtained at 1550 nm. At 1050 and 1550 nm, the *β* of IT-0.0 is ∼4 and ∼10 times larger than IT-8.0, respectively. Therefore, the IT-0.0 was selected as the most suitable saturable absorber for the pulsed laser experiment.

**Table 1:** Calculation of *β* and Im*χ*^(3)^ for all ITO samples.

Samples	IT-0.0	IT-0.5	IT.8.0	IT-0.0-400	IT-0.5-400	IT-8.0-400
**@ 1050 nm**						
*β* (cm/GW)	−50.56	−24.49	12.01	−14.12	−15.19	23.52
Im*χ*^*(*3)^ (×10^−14^ e.s.u.)	37.82	18.32	−8.98	10.57	11.36	−17.60
**@ 1550 nm**						
*β* (cm/GW)	−64.50	−12.90	6.25	4.24	12.60	19.40
Im*χ*^*(3)*^ (×10^−14^ e.s.u.)	41.90	8.39	−4.06	−2.76	−8.19	−12.60

**Figure 5:**
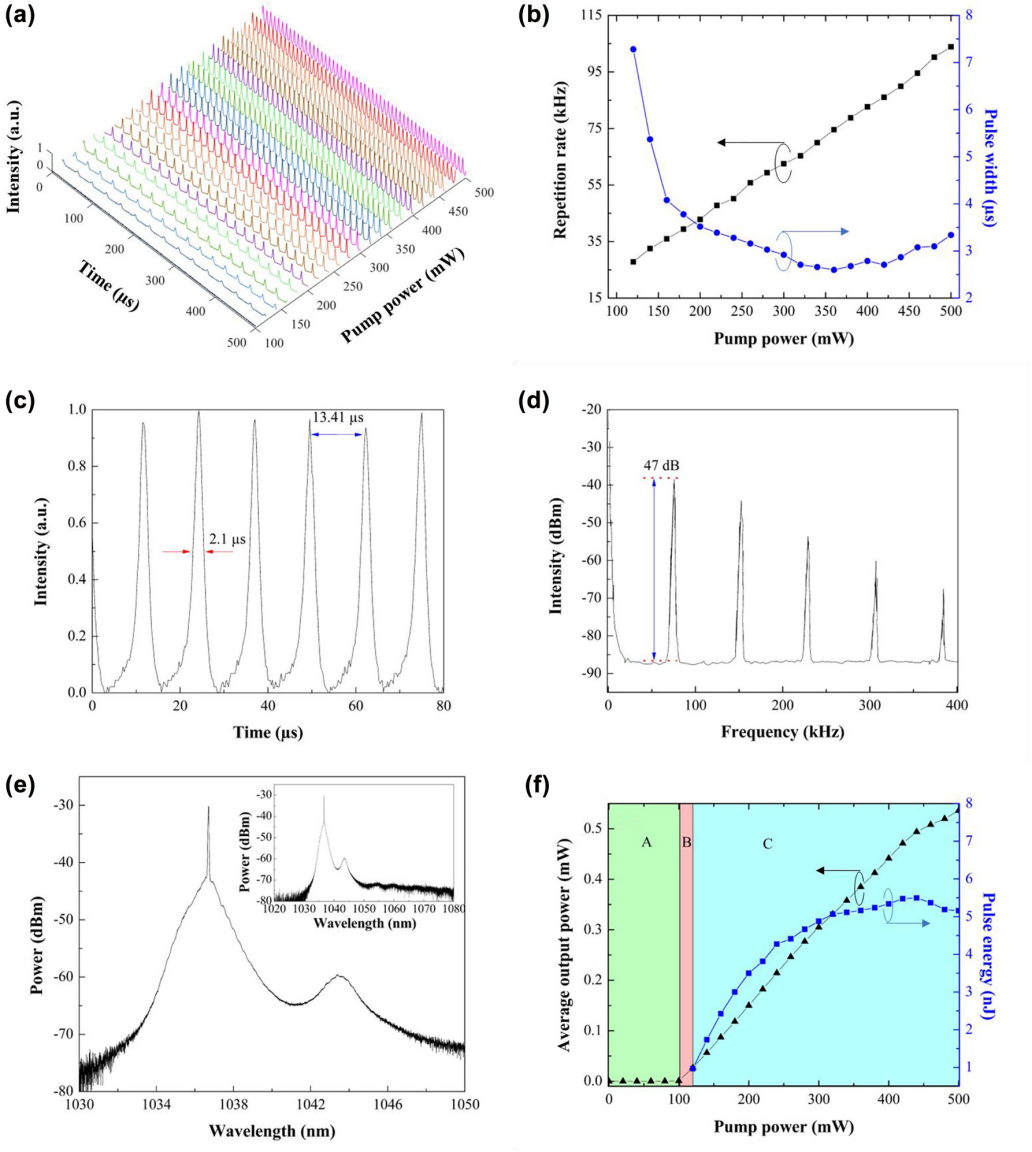
Q-switched ytterbium-doped fiber laser. (a) Evolution of pulse train. (b) Repetition rate and pulse width against pump power. (c) Pulse train, (d) RFS, and (e) optical spectrum of the Q-switched laser at 360 mW pump power. (f) Average output power and pulse energy against pump power.

We employed the sample IT-0.0 for the generation of pulsed fiber laser at ∼1 and ∼1.5 μm. The ∼1 μm laser pulses were generated by integrating the ITO film (deposited onto a thin quartz slide) as saturable absorber into an ytterbium-doped fiber laser cavity. The pulse train of the Q-switched laser was attained by connecting the laser output to an oscilloscope through a photodetector. The evolution of pulse train as a function of pump powers is illustrated in Figure 5(a). The intensities for all pulse train measurement were normalized. The Q-switched pulse train was observed at a pump power threshold of 120 mW and the Q-switching behavior was maintained until 500 mW. The intensity of the Q-switched laser pulse train achieves a plateau at 360 mW which was then gradually reduced at higher pump power. The Q-switched operation vanishes at pump power higher than 500 mW, which was also observed by continuously increasing the pump power beyond the maximum value as reported in ref. [23]. This is because under higher pump power, the SA reaches its saturation limit and the Q-switched pulse train became unstable [24, 25]. The saturation limit of our proposed ITO-SA was observed at the pump power of ∼360 mW for Q-switched YDFL with rather minor changes in pulse width at even higher pump power [23, 26]. By operating the Q-switched YDFL at the pump power from 120 to 500 mW, the repetition rates were tunable with an acceptable range of 76.05 kHz, which is wider than many other recently reported Q-switched fiber lasers generated either through active [27] or passive approaches [28, 29]. The development of repetition rate and pulse width of the Q-switched laser is depicted in Figure 5(b). The repetition rate exhibits an almost linear dependence on pumping power from 27.85 to 103.90 kHz by increasing pump power from 120 to 500 mW. On the other hand, the pulse width reduces from 6.78 to 2.10 μs when the pump power increases from 120 to 360 mW. This measurement matches with typical Q-switching laser behavior. By increasing the pump power from 360 to 500 mW, the pulse width gradually increases from 2.10 to 2.84 μs. This phenomenon is attributed to the gradually reduced intensity of pulse train which vanishes at pump power higher than 500 mW. Therefore, the overall characteristics of the Q-switched laser at the pump power of 360 mW were thoroughly analyzed in Figure 5(c)–(e), owing to its optimized parameters such as the highest intensity and shortest pulse width. The pulse-to-pulse separation of 13.41 μs corresponds to its reciprocal value of pulse repetition rate, *f* = 74.58 kHz. The signal-to-noise ratio (SNR) of the pulsed laser was measured with a radio frequency spectrum (RFS) analyzer. The RFS was measured at the finest bandwidth of the device, 1 kHz for resolution bandwidth and 30 Hz for video bandwidth. At the first peak of 74.58 kHz, the SNR of ∼47 dB was measured between the difference from the peak intensity to the background noise. The optical spectrum of the Q-switched laser was measured at the center wavelength and 3 dB spectral bandwidth were measured as 1036.7 and 0.06 nm, respectively. The Q-switched laser is characterized by the widening of the laser spectral beneath the parasitic continuous wave laser from ∼1034 to 1041 nm. The inset of Figure 5(e) indicates the absence of laser emission at ∼1060 nm. The optical spectrum of the Q-switched fiber laser shows the side peaks located at ∼1042–1045 nm. The possible reason behind this phenomenon is the contribution of optical loss mainly from the ITO-SA. For instance, this side peak emission is expected to be eliminated provided our proposed ITO-SA has a higher optical loss, leaving only the main Q-switched laser emission from ∼1034 to 1041 nm. On the other hand, the Q-switched laser emission from ∼1042 to 1045 nm is also expected to be attained if our proposed ITO-SA has a lower optical loss. The development of average output power and pulse energy as a function of pump power is depicted in Figure 5(f). Region A, B and C denotes the operational status of spontaneous emission, continuous wave lasing and Q-switched lasing, respectively. The continuous wave and Q-switched lasers were generated at pump power threshold of 100 and 120 mW, respectively. The average output power increases from 0.027 to 0.536 mW with a slope efficiency of 0.14% by controlling the pump power from 120 to 500 mW. The pulse energy of the Q-switched laser is calculated by dividing the average output power to pulse repetition rate. The maximum pulse energy was recorded as ∼5.5 nJ at the pump power of 440 mW. Here, we compare our Q-switched ytterbium-doped fiber laser to previous works using different SA materials in Table S1. In contrast to previous works, the Q-switched YDFL using ITO-SA shows wide repetition rate tuning range from 27.85 to 103.90 kHz, which is comparable to refs. [27, 30]. In addition, the SNR of 47 dB is considered excellent, which is slightly lower than refs. [29, 31].

**Figure 6:**
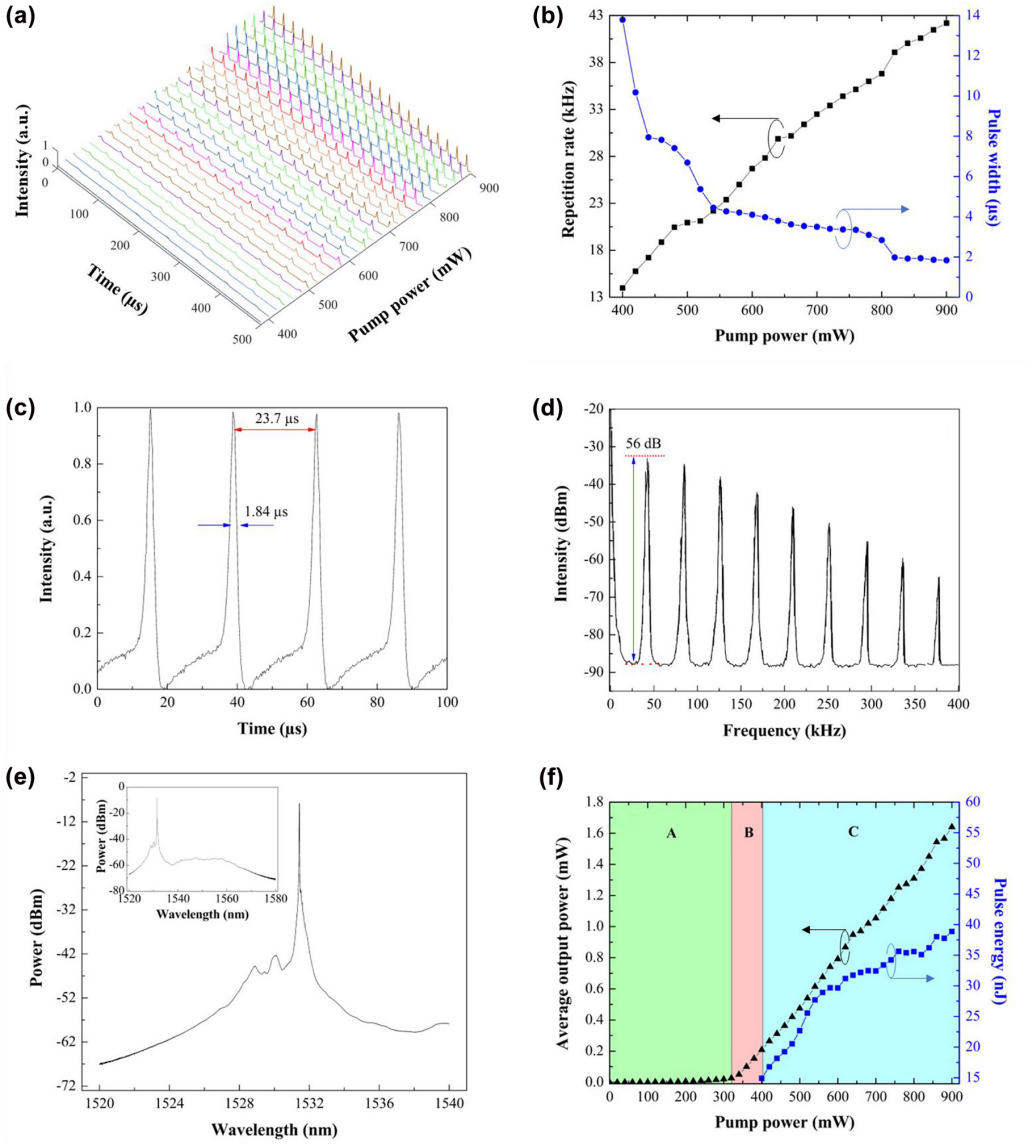
Q-switched erbium-doped fiber laser. (a) Evolution of pulse train. (b) Repetition rate and pulse width against pump power. (c) Pulse train, (d) RFS, and (e) optical spectrum of the Q-switched laser at 900 mW pump power. (f) Average output power and pulse energy against pump power.

Another ∼1.5 μm pulse fiber laser was constructed by integrating the same ITO-based saturable absorber into an erbium-doped fiber laser cavity. The evolution of pulse train as a function of pump powers is plotted in Figure 6(a). The intensities for all pulse train measurement were normalized. The experiment was conducted with a pump power from 380 to 900 mW. The Q-switched laser was initiated at a pump power threshold of 400 mW. The relatively higher pump power threshold here in contrast to Q-switched ytterbium-doped fiber laser is attributed to higher optical loss of IT-0.0 at 1550 nm. Without the SA, the continuous wave laser of the erbium-doped fiber laser cavity was initiated at a pump power threshold of 160 mW. The Q-switched laser possesses closer pulse-to-pulse separation, narrower pulse width and higher intensity at higher pump power. The evolution of repetition rate and pulse width as a function of pump power is depicted in Figure 6(b). The repetition rate evolves from 13.99 to 42.19 kHz and pulse width declines from 13.79 to 1.84 μs by adjusting the pump power from 400 to 900 mW. The characteristics of the Q-switched laser at the maximum pump power were thoroughly analyzed in Figure 6(c)–(e). The peak intensity of the Q-switched laser pulse train was maintained at a rather horizontal level. The pulse-to-pulse separation of 23.7 μs corresponds to its reciprocal value of pulse repetition rate, *f* = 42.19 kHz. The RFS was measured at the finest resolution setting, 1 kHz for resolution bandwidth and 30 Hz for video bandwidth. The SNR was measured as 56 dB which validates the excellent laser stability. The center wavelength and 3 dB spectral bandwidth of the Q-switched laser were measured from the optical spectrum as 1531.43 and 0.02 nm, respectively. The broader spectral bandwidth underneath the peak wavelength from −47 to −27 dBm of optical power exhibits the characteristic of Q-switched laser. The inset of Figure 6(e) shows that the Q-switched laser was solely observed at the emission of ∼1530 nm. The optical spectrum of the Q-switched laser at this wavelength region also shows the side peaks, which could be owing to the optical loss mainly contributed by the ITO-SA. The side peaks could be eliminated by employing an ITO-SA with either higher optical loss with emission at shorter wavelength or lower optical loss with emission at longer wavelength. Sometimes, the side peaks could be eliminated by adjusting the polarization controller in the laser cavity. The side peak profiles of Q-switched fiber laser were also observed in refs. [32, 33]. In fact, the broadening of the optical spectrum is more important than the side peak emission because it outlines the main characteristic for Q-switched fiber laser. In previous works, the side peaks of Q-switched fiber lasers were suppressed by employing filters, i.e., fiber Bragg gratings (FBGs) and tunable wavelength filter, to generate single wavelength [34], dual wavelength [35] or C-band and L-band Q-switched fiber laser emissions [36]. The evolution of average output power and pulse energy as a function of pump power is presented in Figure 6(f). Region A, B and C denotes the operational status of spontaneous emission, continuous wave lasing, and Q-switched lasing, respectively. The continuous wave and Q-switched lasers were initiated at pump power threshold of 320 and 400 mW, respectively. At region B and C, the average output power evolves with ∼28 μW for every 10 mW increased in pump powers. At region C, the pulse energy develops from 14.9 to 38.9 nJ by increasing the pump power from 400 to 900 mW. The maximum average output power and pulse energy were recorded as 1.64 mW and 38.87 nJ at the pump power of 900 mW. Table S2 compares the Q-switched erbium-doped fiber laser using different SA materials. Here, we notice that our ITO SA has comparable Q-switching performance to previous works in terms of frequency, pulse width, output power and pulse energy. Nevertheless, this work presents the highest SNR of 56 dB employing the ITO-SA, which shows the high stability of our ITO-SA for erbium-doped fiber laser.

The difference in the conditions to realize passive mode-locking and passive Q-switching is that the requirement for modulation depth of SA for Q-switching usually should be larger than that for mode-locking to change the cavity loss [10, 37]. Mode-locking could be realized when the SA reaches the minimum modulation depth for stable fiber lasers [38]. There are several methods to improve the modulation depth. For instance, it can be enhanced by scribing a polymer film on the end facet of a fiber connector before the ITO film [39] and by reducing the thickness or concentration of the ITO used in the fiber laser [40]. Moreover, the response time of the SA for Q-switching should be much shorter than the lifetime of doped ions (in the gain medium) on the metastable level whereas the response time of the SA for mode-locking should be shorter than round-trip time [41]. Therefore, the ITO-SA is feasible to realize the mode-locking operation with much optimized parameters on these circumstances.

## 4 Conclusions

We have fabricated a group of ITO thin films with ∼2 μm thickness by magnetron sputtering under controlled deposition conditions. The ITO thin film obtained under optimized condition shows a broad ENZ region of 885–2060 nm. The third-order nonlinear absorption coefficient, *β* and imaginary part of third-order nonlinear susceptibility, Im*χ*^(3)^ of the ITO thin films were controllable with the growth conditions. The highest *β* and Im*χ*^(3)^ were measured as −64.5 cm/GW and ∼45 × 10^−14^ e.s.u., respectively, for the ITO thin film fabricated under low oxygen content condition (O_2_:Ar ratio of 0.0) before annealing. The strong saturable absorption of the ITO thin film in the NIR regions was utilized in the development of optical switches for generation of nJ laser pulses at ∼1 and ∼1.5 μm through the Q-switching method in fiber lasers. Our results show that ITO thin films with tunable NLO response have great potential for applications as NIR optical switches.

## Supplementary Material

Supplementary Material Details
