# Supplementary material for: Tunable optical nonlinearity of indium tin oxide for optical switching in epsilon-near-zero region
Source: Nanophotonics. 2022 Aug 9;11(18):4209–19. doi: 10.1515/nanoph-2022-0306 (PMC11501465; doi:10.1515/nanoph-2022-0306)
Supplement: Supplementary file 1 — Supplementary Material Details [file nanoph-2022-0306_suppl.docx]

Supplementary Materials

Tunable optical nonlinearity of indium tin oxide for optical switching in epsilon-near-zero region

Kuen Yao Lau, Yuting Yang, Di Zhao, Xiaofeng Liu* and Jianrong Qiu*

Pulsed laser experiment

A section of rare-earth gain fiber, OFS YDF-350 and LIEKKI Er 110-4/125 were pumped by a 980 nm laser diode (LD) via a wavelength division multiplexer (WDM) in the ~1.0 and ~1.5 μm fiber lasers. An isolator ensures unidirectional signal propagation of the laser cavity in clockwise direction. The output laser was measured through the 10% signal siphoned from a 90:10 optical coupler whereas the remaining 90% signal reverts to the laser cavity. The polarization state was adjusted by a polarization controller. All components are polarization insensitive which avoids the possibilities of pulse-induced nonlinear polarization rotation. At the initial stage, the laser cavity was examined without the saturable absorber. This is to validate that the laser pulses are solely contributed by the IT-0.0 saturable absorber.


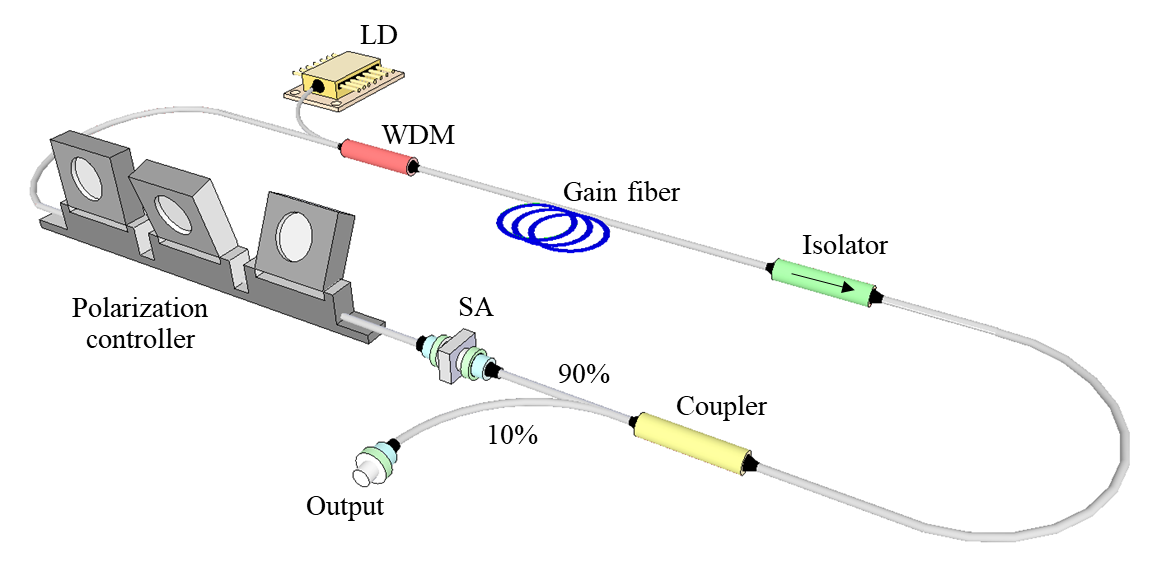


**Fig. S1.** Schematic diagram of fiber laser cavity.

**Material Characterizations for ITO thin film**

The surface topography and thickness of the ITO samples deposited on quartz substrate were observed by a scanning electron microscope as illustrated in Figs. S2 & S3. These ITO samples show closely-packed grain profiles with average sizes of ~0.1-1 μm before annealing and ~0.1-0.3 μm after annealing. Therefore, both O_2_:Ar ratio and heat treatment exhibit influence during ITO sample preparation on the surface topography of the ITO samples. All the ITO thin films are uniformly densified and firmly attached to the quartz substrate. The absence of macro-defects such as pinholes existing between the grains validates the high quality of the synthesized ITO samples. The thicknesses of all ITO thin films are ~2 μm, based on Fig. S3.


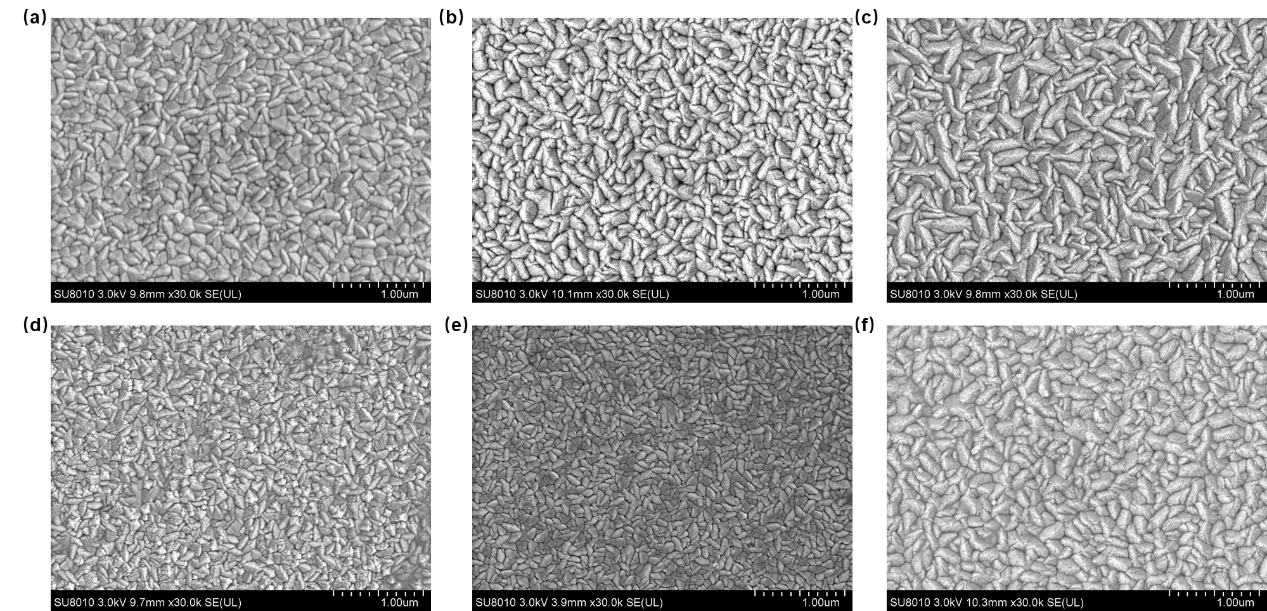


**Fig. S2.** SEM images for ITO thin films: (a) IT-0.0, (b) IT-0.5, IT-8.0 before annealing, (d) IT-0.0-400, (e) IT-0.5-400 and (f) IT-8.0-400 after annealing.


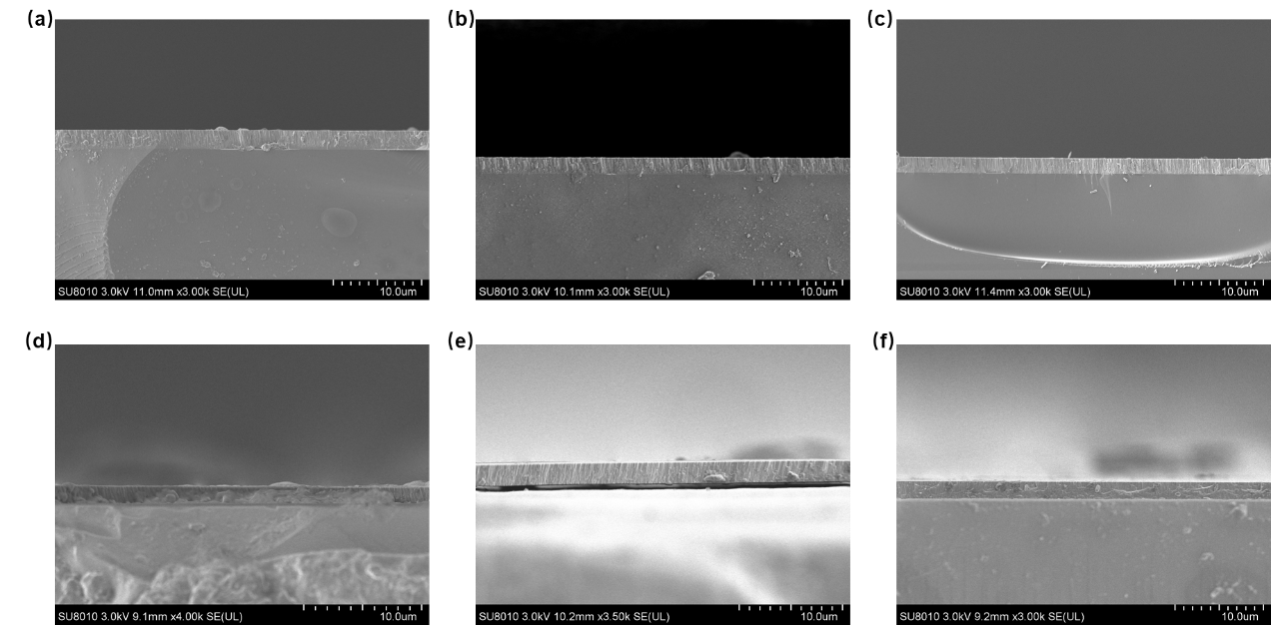


**Fig. S3.** SEM images in cross-sectional view for ITO thin films on quartz substrates: (a) IT-0.0, (b) IT-0.5, IT-8.0 before annealing, (d) IT-0.0-400, (e) IT-0.5-400 and (f) IT-8.0-400 after annealing.

The chemical composition in the ITO thin films on quartz substrate was characterized by an X-ray photoelectron spectrum (XPS) as presented in Fig. S4. Fig. S4(a) shows the strongest peak of In-3d, as well as other peaks featuring the Sn-3d, O-1s and In-3p. Figs. S4(b) and S4(c) present the corresponding In-3d and Sn-3d peaks with high resolution extracted from the full XPS. While Raman spectrum reveals only a slight change in the peak position and intensity, the high resolution XPS spectra exhibit a clear shift of In 3d from ~452 eV (In-3d_3/2_) to ~444 eV (In-3d_5/2_) and Sn 3d from ~495 eV (Sn-3d_3/2_) to ~486 eV (Sn-3d_5/2_). The binding energies of In-3d_5/2_ and Sn-3d_5/2_ at 443.68 eV and 485.68 eV confirm the oxidation states of the In^3+^ and Sn^4+^, respectively [1].


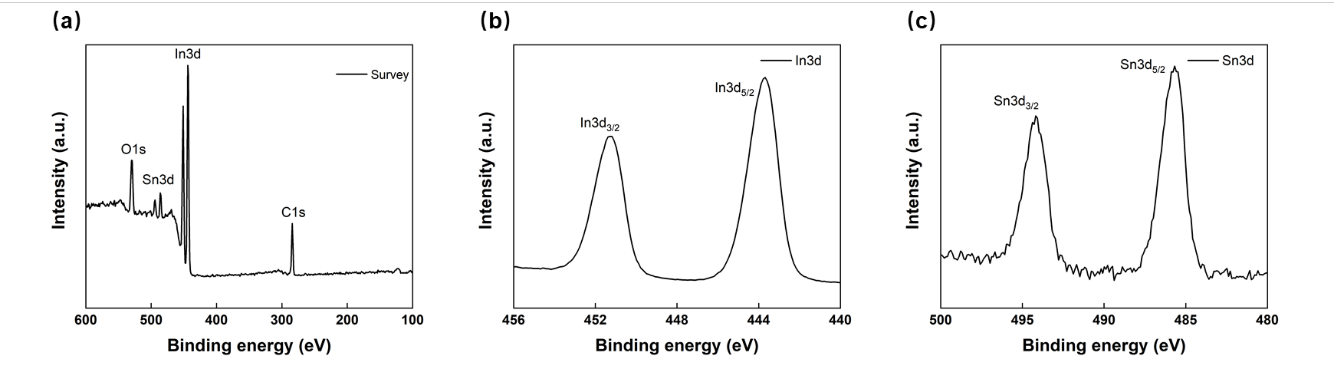


**Fig. S4.** XPS spectrum for ITO thin film on quartz substrates: (a) Full spectrum. High resolution spectra in (b) In-3d and (c) Sn-3d regions.

**Calculation of real part permittivity**

The Drude-Lorentz model was used to describe the plasmonic resonance absorption [2,3]. The complex dielectric constant, ε(*ω*) of the ITO thin film can be expressed in terms of the angular frequency by:

$\varepsilon_{\omega}=\varepsilon_{\infty}-\frac{\omega_{p}^{2}}{\omega^{2}+i\omega\Gamma}$ (1)

where ε_∞_ is the asymptotic value for ITO (ε_∞_ = 1.46), *ω_p_* is the plasma angular frequency (*ω_p_* = 1.51 × 10^15^ rad/s), *ω* is the angular frequency (*ω* = 2πc/λ) and *Γ* is the damping rate (*Γ* = 2.98 × 10^14^ rad/s) [4]. The reciprocal of *Γ* is the average relaxation time, τ:

$\tau=\frac{1}{\Gamma}$ (2)

where τ is approximately 3.36 × 10^-15^ seconds. By solving Equation (1), the real (ε_r_) and imaginary (ε_i_) parts of the permittivity of the materials are expressed as:

$\varepsilon_{r}=\varepsilon_{\infty}-\frac{\omega_{p}^{2}\times\tau^{2}}{1+\omega^{2}\tau^{2}}$ (3)

$\varepsilon_{i}=\frac{\omega_{p}^{2}\tau/\omega}{1+\omega^{2}\tau^{2}}$ (4)

The refractive index (*n*) and extinction coefficient (*k*) of the materials are expressed as:

$n=\sqrt{\frac{1}{2}(\varepsilon_{r}^{2}+\varepsilon_{i}^{2})^{\frac{1}{2}}+\frac{\varepsilon_{r}}{2}}$ (5)

$k=\sqrt{\frac{1}{2}(\varepsilon_{r}^{2}+\varepsilon_{i}^{2})^{\frac{1}{2}}-\frac{\varepsilon_{r}}{2}}$ (6)

**Calculation of the nonlinear saturable absorption coefficient and third-order nonlinear susceptibility**

The nonlinear saturable absorption coefficient (*β*) is calculated according to the nonlinear absorption theory as [5]:

$\alpha=\alpha_{0}+\beta I$ (7)

where *α* is absorption coefficient, *α*_0_ is linear absorption coefficient and *I* is incident intensity. The *α*_0_ is given by Lambert’s formula:

$T=e^{-\alpha_{0}L}$ (8)

The open aperture Z-scan curves are fitted according to the nonlinear absorption model:

$T(z)=\frac{\ln(1+q_{0}(z))}{q_{0}(z)}$ (9)

where T(z) is the normalized transmittance recorded as the Y-axis of the Z-scan curves and *q*_0_(*z*) describes the effective thickness of the ITO samples, L_eff_:

$q_{0}(z)=\frac{\beta I_{0}L_{eff}}{(1+\frac{z^{2}}{z_{0}^{2}})}=\frac{1-e^{-\alpha_{0}L}}{\alpha_{0}}$ (10)

where z_0_ is the diffraction length of the beam (πω_0_^2^/λ), ω_0_ is the beam waist radius and L is the sample thickness. The imaginary part of third order susceptibility (*Imχ^(3)^*) is calculated as:

$\mathrm{Im}\chi^{(3)}=\frac{(10^{-7}c\lambda n^{2})\beta}{96\pi^{2}}$ (11)

where *c* is the speed of light and *n* is the refractive index of the material.

**Comparison of Q-switched ytterbium-doped and erbium-doped fiber lasers.**

The comparison of Q-switched fiber laser at ~1 μm and ~1.5 μm with our proposed ITO SA to different SA materials was performed in Table S1 and Table S2, respectively. The PP, λ_c_, *f*, τ, OP, PE and SNR are abbreviated as pump power, center wavelength, pulse repetition rate, pulse width, output power, pulse energy and signal-to-noise ratio, respectively.

**Table S1.** Comparison of Q-switched ytterbium-doped fiber laser using different SA materials.

| **Materials** | **PP (mW)** | **λ_c_ (nm)** | ***f* (kHz)** | **τ (μs)** | **OP (mW)** | **PE (nJ)** | **SNR (dB)** | **Ref.** |
| --- | --- | --- | --- | --- | --- | --- | --- | --- |
| Graphene | 382.4 | 1060 | 30.32-101.29 | 2.16 | 0.99 | ~31 (Highest) | - | [6] |
| rGO-Ag nanoparticles | 78.97-158.60 | 1044.4 | 52.33-62.10 | 2.48-1.10 | 0.10-0.22 | 1.88-3.52 | ~56 | [7] |
| Ag nanoparticles | 210-600 | 1033.3 | 66.6-184.8 | 3.93-1.01 | 1.46-10.77 | 21.9-58.3 | ~30 | [8] |
| MoS_2_ | - | 1030-1070 | 65.3-89.0 | 4.40-2.68 | 7.0-10.5 | 126 (Highest) | ~45 | [9] |
| PtTe_2_ | 118-146.5 | 1066 | 23.0-33.5 | 13.6-5.2 | 1.03-2.48 | ~74 (Highest) | 35 | [10] |
| Bi_2_Se_3_ | 42.5-106.2 | 1067.66 | 8.3-29.1 | 8.3-1.95 | ~0.01-0.46 | 21  41.3 (Highest) | 48 | [11] |
| Black Phosphorus | 100-210 | 1064.7 | 8.9-32.3 | 14.18-4.93 | 14.54 (Highest) | 450.14 | ~35 | [12] |
| Semiconducting polymer dot | 124 | 1036 | 30.59 | 4.2 | 2.74 | 89.57 | - | [13] |
| ITO | 120-500 | 1036.7 | 27.85-103.90 | 6.78-2.10 | 0.027-0.536 | ~5.5 (Highest) | 47 | This work |

**Table S2.** Comparison of Q-switched erbium-doped fiber laser using different SA materials.

| **Materials** | **PP (mW)** | **λ_c_ (nm)** | ***f* (kHz)** | **τ (μs)** | **OP (mW)** | **PE (nJ)** | **SNR (dB)** | **Ref.** |
| --- | --- | --- | --- | --- | --- | --- | --- | --- |
| rGO | 120-175 | ~1563 | 104-116 | 3.85-1.85 | ~9.2-14.6 | 90-125 | - | [14] |
| Cabon nanocage | 76-220 | 1569.3 | 58.5 | 3.3 | 0.18-7.39 | 126.3 (Highest) | - | [15] |
| NiPS_3_ | 40-105 | ~1560 | 20.6-33.9 | 10.24-4.64 | ~1.05 | 31.01 (Highest) | 45.54 | [16] |
| MoS_2_ | 18.9-227.1 | 1551.2 | 8.77-43.47 | 26.7-3.3 | 5.91 (Highest) | 160 | 50 | [17] |
| MoS_2_ | 371-470 | 1549.83 | 116-131 | 760-660 | 20 | 152 | - | [18] |
| WS_2_ | 30-540 | 1560 | 29.5-367.8 | 1269-154.9 | ~1-25.2 | ~10-68.5 | 42 | [19] |
| WTe_2_ | 124.9-244.5 | 1522-1578 | 38.39-55.56 | 3.67-1.77 | 0.28-1.01 | 7.31-18.09 | 48.5 | [20] |
| PtS_2_ | 53-84 | 1568.8 | 18.1-24.6 | 9.6-4.2 | 1.1 (Highest) | 45.6 | - | [21] |
| Bi_2_Se_3_ | 96-360 | 1565 | 459-940 | 7.76-1.9 | ~3-22.35 | ~8-23.8 | 50 | [22] |
| NiS_2_ | 37-200 | 1561.86 | 195.3-243.9 | 822-237 | ~6-30.2 | ~1230 | - | [23] |
| BP | 50-185 | 1562.9 | 6.89-15.47 | 39.84-10.32 | ~1.46 | 94.3 (Highest) | ~45 | [24] |
| Gold nanosheet | 237 | 1562 | 58.1 | 1.78 | 7.7 | 133 | - | [25] |
| CuS nanocrystal | 1400-5600 | 1567.2 | 16.6-51.14 | 8.7-2.0 | 0.3-61.1 | ~18.07-1195 | - | [26] |
| Tellurene | 46-195 | 1563.7 | 15.92-47.61 | 8.915-5.196 | 1.63-8.134 | ~102.39-170.85 | 44 | [27] |
| Semiconducting polymer dot | 34-118 | 1561 | 12.76-26.4 | 20.81-8.02 | 0.35-2.74 | 27.43-103.97 | - | [13] |
| ITO | 400-900 | 1531.43 | 13.99-42.19 | 13.78-1.84 | ~0.2-1.64 | 14.9-38.9 | 56 | This work |

**References**

[1] G. Folcher, H. Cachet, M. Froment, and J. Bruneaux, “Anodic corrosion of indium tin oxide films induced by the electrochemical oxidation of chlorides,” *Thin Solid Films*, vol. 301, pp. 242–248, 1997. [https://doi.org/10.1016/S0040-6090(97)00024-2](https://doi.org/10.1109/map.2012.6230714)

[2] J. M. Luther, P. K. Jain, T. Ewers, and A. P. Alivisatos, “Localized surface plasmon resonances arising from free carriers in doped quantum dots,” *Nat. Mater.*, vol. 10, pp. 361–366, 2011. [https://doi.org/10.1038/nmat3004](https://doi.org/10.1109/map.2012.6230714)

[3] Q. Guo, Y. Cui, Y. Yao, Y. Ye, Y. Yang, X. Liu, S. Zhang, X. Liu, J. Qiu, and H. Hosono, “A Solution-Processed Ultrafast Optical Switch Based on a Nanostructured Epsilon-Near-Zero Medium,” *Adv. Mater.*, vol. 29, p. 1700754, 2017. [https://doi.org/10.1002/adma.201700754](https://doi.org/10.1109/map.2012.6230714)

[4] A. B. Kuzmenko, “Kramers-Kronig constrained variational analysis of optical spectra,” *Rev. Sci. Instrum.*, vol. 76, p. 083108, 2005. [https://doi.org/10.1063/1.1979470](https://doi.org/10.1109/map.2012.6230714)

[5] A. O. Mousa, Y. H. Kadhim, and B. A. Naser, “Study of Nonlinear Optical Properties of Nematic Liquid Crystal Materials,” *Int. J. Phys. Res.*, vol. 6, pp. 17–24, 2016. [https://www.researchgate.net/publication/340998037](https://doi.org/10.1109/map.2012.6230714)

[6] A. Ren, M. Feng, F. Song, Y. Ren, S. Yang, Z. Yang, Y. Li, Z. Liu, and J. Tian, “Actively Q-switched ytterbium-doped fiber laser by an all-optical Q-switcher based on graphene saturable absorber,” *Opt. Express*, vol. 23, no. 16, pp. 21490–21496, 2015. [https://doi.org/10.1364/oe.23.021490](https://doi.org/10.1109/map.2012.6230714)

[7] H. Ahmad, H. S. Albaqawi, N. Yusoff, S. A. Reduan, and C. W. Yi, “Reduced Graphene Oxide-Silver Nanoparticles for Optical Pulse Generation in Ytterbium- and Erbium-Doped Fiber Lasers,” *Sci. Rep.*, vol. 10, p. 9408, 2020. [https://doi.org/10.1038/s41598-020-66253w](https://doi.org/10.1109/map.2012.6230714)

[8] B. Fu, P. Wang, Y. Li, M. Condorelli, E. Fazio, J. Sun, L. Xu, V. Scardaci, and G. Compagnini, “Passively Q-switched Yb-doped all-fiber laser based on Ag nanoplates as saturable absorber,” *Nanophotonics*, vol. 9, no. 12, pp. 3873–3880, 2020. [https://doi.org/10.1515/nanoph-2020-0015](https://doi.org/10.1109/map.2012.6230714)

[9] R. I. Woodward, E. J. R. Kelleher, R. C. T. Howe, G. Hu, F. Torrisi, T. Hasan, S. V. Popov, and J. R. Taylor, “Tunable Q-switched fiber laser based on saturable edge-state absorption in few-layer molybdenum disulfide (MoS_2_),” *Opt. Express*, vol. 22, no. 25, pp. 31113–31122, 2014. [https://doi.org/10.1364/oe.22.031113](https://doi.org/10.1109/map.2012.6230714)

[10] P. K. Cheng, C. Y. Tang, X. Y. Wang, S. Ma, H. Long, and Y. H. Tsang, “Passively Q-switched Ytterbium-doped fiber laser based on broadband multilayer Platinum Ditelluride (PtTe_2_) saturable absorber,” *Sci. Rep.*, vol. 9, p. 10106, 2019. [https://doi.org/10.1038/s41598-019-46658-y](https://doi.org/10.1109/map.2012.6230714)

[11] Z. Luo, Y. Huang, J. Weng, H. Cheng, Z. Lin, B. Xu, Z. Cai, and H. Xu, “1.06 μm Q-switched ytterbium-doped fiber laser using few-layer topological insulator Bi_2_Se_3_ as a saturable absorber,” *Opt. Express*, vol. 21, no. 24, pp. 29516–29522, 2013. [https://doi.org/10.1364/oe.21.029516](https://doi.org/10.1109/map.2012.6230714)

[12] T. Wang, X. Jin, J. Yang, J. Wu, Q. Yu, Z. Pan, H. Wu, J. Li, R. Su, J. Xu, K. Zhang, T. He, and P. Zhou, “Ultra-stable pulse generation in ytterbium-doped fiber laser based on black phosphorus,” *Nanoscale Adv.*, vol. 1, pp. 195–202, 2019. [https://doi.org/10.1039/c8na00221e](https://doi.org/10.1109/map.2012.6230714)

[13] H. Chen, F. Wang, M. Qian, X. Zhou, Z. Li, T. Cheng, and G. Qin, “Semiconducting polymer dots as broadband saturable absorbers for Q-switched fiber lasers,” *J. Mater. Chem. C*, vol. 8, pp. 4919–4925, 2020. [https://doi.org/10.1039/c9tc06599g](https://doi.org/10.1109/map.2012.6230714)

[14] G. Sobon, J. Sotor, J. Jagiello, R. Kozinski, K. Librant, M. Zdrojek, L. Lipinska, and K. M. Abramski, “Linearly polarized, Q-switched Er-doped fiber laser based on reduced graphene oxide saturable absorber,” *Appl. Phys. Lett.*, vol. 101, p. 241106, 2012. [https://doi.org/10.1063/1.4770373](https://doi.org/10.1109/map.2012.6230714)

[15] X. Xu, J. Chen, L. Sun, S. Chu, D. Sun, J. Lu, D. Wang, and S. Ruan, “Low-temperature synthesis of a carbon nanocage saturable absorber for pulsed erbium-doped fiber laser generation,” *J. Mater. Chem. C*, vol. 10, pp. 235–243, 2022. [https://doi.org/10.1039/D1TC04791D](https://doi.org/10.1109/map.2012.6230714)

[16] J. Wang, T. Wang, X. Shi, J. Wu, Y. Xu, X. Ding, Q. Yu, K. Zhang, P. Zhou, and Z. Jiang, “NiPS_3_ nanosheets for passive pulse generation in an Er-doped fiber laser,” *J. Mater. Chem. C*, vol. 7, pp. 14625–14631, 2019. [https://doi.org/10.1039/C9TC04722K](https://doi.org/10.1109/map.2012.6230714)

[17] Y. Huang, Z. Luo, Y. Li, M. Zhong, B. Xu, K. Che, H. Xu, Z. Cai, J. Peng, and J. Weng, “Widely-tunable, passively Q-switched erbium-doped fiber laser with few-layer MoS_2_ saturable absorber,” *Opt. Express*, vol. 22, no. 21, pp. 25258–25266, 2014. [https://doi.org/10.1364/oe.22.025258](https://doi.org/10.1109/map.2012.6230714)

[18] J. Ren, S. Wang, Z. Cheng, H. Yu, H. Zhang, Y. Chen, L. Mei, and P. Wang, “Passively Q-switched nanosecond erbium-doped fiber laser with MoS_2_ saturable absorber,” *Opt. Express*, vol. 23, no. 5, pp. 5607–5613, 2015. [https://doi.org/10.1364/oe.23.005607](https://doi.org/10.1109/map.2012.6230714)

[19] H. Chen, Y. Chen, J. Yin, X. Zhang, T. Guo, and P. Yan, “High-damage-resistant tungsten disulfide saturable absorber mirror for passively Q-switched fiber laser,” *Opt. Express*, vol. 24, no. 15, pp. 16287–16296, 2016. [https://doi.org/10.1364/oe.24.016287](https://doi.org/10.1109/map.2012.6230714)

[20] H. Ahmad, H. S. Albaqawi, N. Yusoff, and C. W. Yi, “56 nm Wide-Band Tunable Q-Switched Erbium Doped Fiber Laser with Tungsten Ditelluride (WTe_2_) Saturable Absorber,” *Sci. Rep.*, vol. 10, p. 9860, 2020. [https://doi.org/10.1038/s41598-020-66664-9](https://doi.org/10.1109/map.2012.6230714)

[21] X. Wang, P. K. Cheng, C. Y. Tang, H. Long, H. Yuan, L. Zeng, S. Ma, W. Qarony, and Y. H. Tsang, “Laser Q-switching with PtS_2_ microflakes saturable absorber,” *Opt. Express*, vol. 26, no. 10, pp. 13055–13060, 2018. [https://doi.org/10.1364/oe.26.013055](https://doi.org/10.1109/map.2012.6230714)

[22] Z. Yu, Y. Song, J. Tian, Z. Dou, H. Guoyu, K. Li, H. Li, and X. Zhang, “High-repetition-rate Q-switched fiber laser with high quality topological insulator Bi_2_Se_3_ film,” *Opt. Express*, vol. 22, no. 10, pp. 11508–11515, 2014. [https://doi.org/10.1364/oe.22.011508](https://doi.org/10.1109/map.2012.6230714)

[23] S. Li, Y. Yin, Q. Ouyang, G. Ran, Y. Chen, E. Lewis, G. Farrell, M. Tokurakawa, S. W. Harun, and P. Wang, “Nanosecond passively Q-switched fibre laser using a NiS_2_ based saturable absorber,” *Opt. Express*, vol. 27, no. 14, pp. 19843–19851, 2019. [https://doi.org/10.1364/oe.27.019843](https://doi.org/10.1109/map.2012.6230714)

[24] Y. Chen, G. Jiang, S. Chen, Z. Guo, X. Yu, C. Zhao, H. Zhang, Q. Bao, S. Wen, D. Tang, and D. Fan, “Mechanically exfoliated black phosphorus as a new saturable absorber for both Q-switching and mode-locking laser operation,” *Opt. Express*, vol. 23, no. 10, pp. 5989–5992, 2015. [https://doi.org/10.1364/oe.23.012823](https://doi.org/10.1109/map.2012.6230714)

[25] D. Fan, C. Mou, X. Bai, S. Wang, N. Chen, and X. Zeng, “Passively Q-switched erbium-doped fiber laser using evanescent field interaction with gold-nanosphere based saturable absorber,” *Opt. Express*, vol. 22, no. 15, pp. 18537–18542, 2014. [https://doi.org/10.1364/oe.22.018537](https://doi.org/10.1109/map.2012.6230714)

[26] M. Liu, D. Zhou, Z. Jia, Z. Li, N. Li, S. Li, Z. Kang, J. Yi, C. Zhao, G. Qin, H. Song, and W. Qin, “Plasmonic Cu_1.8_S nanocrystals as saturable absorbers for passively Q-switched erbium-doped fiber lasers,” *J. Mater. Chem. C*, vol. 5, pp. 4034–4039, 2017. [https://doi.org/10.1039/C6TC05565F](https://doi.org/10.1109/map.2012.6230714)

[27] W. Zhang, G. Wang, F. Xing, Z. Man, F. Zhang, K. Han, H. Zhang, and S. Fu, “Passively Q-switched and mode-locked erbium-doped fiber lasers based on tellurene nanosheets as saturable absorber,” *Opt. Express*, vol. 28, no. 10, pp. 14729–14739, 2020. [https://doi.org/10.1364/oe.28.392944](https://doi.org/10.1109/map.2012.6230714)
